# Critical role of astrocytic interleukin-17 A in post-stroke survival and neuronal differentiation of neural precursor cells in adult mice

**DOI:** 10.1038/cddis.2015.284

**Published:** 2016-06-23

**Authors:** Y Lin, J-C Zhang, C-Y Yao, Y Wu, A F Abdelgawad, S-L Yao, S-Y Yuan

**Affiliations:** 1 Department of Anesthesia, Institute of Anesthesia and Critical Care, Union Hospital, Tongji Medical College, Huazhong University of Science and Technology, Wuhan 430022, China; 2 Department of Critical Care Medicine, Institute of Anesthesia and Critical Care, Union Hospital, Tongji Medical College, Huazhong University of Science and Technology, Wuhan 430022, China; 3 Department of Neurology, Union Hospital, Tongji Medical College, Huazhong University of Science and Technology, Wuhan 430022, China; 4 Department of Dermatology, Union Hospital, Tongji Medical College, Huazhong University of Science and Technology, Wuhan 430022, China; 5 Department of Anesthesia, Faculty of Medicine, Benha University, Benha, Egypt

## Abstract

The brain and the immune system interact in complex ways after ischemic stroke, and the long-term effects of immune response associated with stroke remain controversial. As a linkage between innate and adaptive immunity, interleukin-17 A (IL-17 A) secreted from gamma delta (γδ) T cells has detrimental roles in the pathogenesis of acute ischemic stroke. However, to date, the long-term actions of IL-17 A after stroke have not been investigated. Here, we found that IL-17 A showed two distinct peaks of expression in the ischemic hemisphere: the first occurring within 3 days and the second on day 28 after stroke. Our data also showed that astrocyte was the major cellular source of IL-17 A that maintained and augmented subventricular zone (SVZ) neural precursor cells (NPCs) survival, neuronal differentiation, and subsequent synaptogenesis and functional recovery after stroke. IL-17 A also promoted neuronal differentiation in cultured NPCs from the ischemic SVZ. Furthermore, our *in vitro* data revealed that in primary astrocyte cultures activated astrocytes released IL-17 A via p38 mitogen-activated protein kinase (MAPK). Culture media from reactive astrocytes increased neuronal differentiation of NSCs *in vitro*. Blockade of IL-17 A with neutralizing antibody prevented this effect. In addition, after screening for multiple signaling pathways, we revealed that the p38 MAPK/calpain 1 signaling pathway was involved in IL-17 A-mediated neurogenesis *in vivo* and *in vitro*. Thus, our results reveal a previously uncharacterized property of astrocytic IL-17 A in the maintenance and augment of survival and neuronal differentiation of NPCs, and subsequent synaptogenesis and spontaneous recovery after ischemic stroke.

Stroke is the major cause of permanent disability in adults worldwide because of the brain's limited capacity for neural repair.^1, 2^ Clinical and research efforts have focused on promoting post-ischemic neurovascular remodeling and functional recovery after stroke. Previous studies have found that many factors can affect the process of neurovascular remodeling after stroke, such as growth factors, neurotrophins, chemokines and immune cytokines.^3^ Among the panel of novel factors, accumulating evidence suggests that cytokines in immune-mediated inflammatory response have key roles in brain regeneration and neurovascular remodeling after stroke.^4, 5^

The elements of the immune system are shown to be intimately involved in all stages of ischemic stroke, from the early damaging events to the late regenerative processes underlying post-ischemic tissue repair, which determinates the fate of the stroke and the survival of stroke patients.^5^ Ischemic cell death activates innate immunity and sets the stage for adaptive immunity. The early activation of innate immunity and the release of immune cytokines exert detrimental effects in acute phase of stroke,^6, 7^ which are not related to adaptive immunity.^8^ However, the adaptive immunity to brain antigens occurs in the later phases and may have important roles in neurovascular remodeling and functional recovery during stroke recovery.^5^ Among various immune cytokines secreted by immune cells, we focused on interleukin-17 A (IL-17 A) owing to three recently proposed ideas. First is the suggestion that IL-17 A is a solid link between innate and adaptive immunity and can exert both deleterious as well as beneficial effects in neuroinflammation.^7, 9, 10^ Second, the crosstalk between central reactive astrocytes and precursor cells during stroke recovery supports neurovascular remodeling and functional recovery.^11^ In neuroinflammatory diseases, IL-17 A is specifically expressed in reactive astrocytes.^10, 12^ Third, it has been proposed that neurovascular mediators triggered after stroke may have a biphasic role in the pathophysiology of stroke; mediators that are deleterious during the acute stage may have beneficial roles in the later phases of stroke recovery.^13^ IL-17 A released from gamma delta (γδ) T cells mediates innate immune response via Toll-like receptor 4, and contributes to post-ischemic brain inflammation and injury in acute stroke.^6, 7^ However, the long-term actions of IL-17 A beyond the acute stroke remain unclear.

In this study, we showed that IL-17 A from reactive astrocytes maintained and augmented the survival and neuronal differentiation of neural precursor cells (NPCs) in the subventricular zone (SVZ), and subsequent synaptogenesis and spontaneous recovery through the p38 mitogen-activated protein kinase (MAPK)/calpain 1 signaling pathway. Therefore, although IL-17 A is well known for its damage role in acute stroke, it may be an essential mediator for ischemia-induced neurorepair and spontaneous recovery after stroke. Our findings reveal a previously unsuspected role for IL-17 A in the survival and neuronal differentiation of NPCs, and subsequent synaptogenesis and long-term functional outcome after ischemic stroke.

## Results

### IL-17A increases during the period of stroke recovery

**Figure 1:**
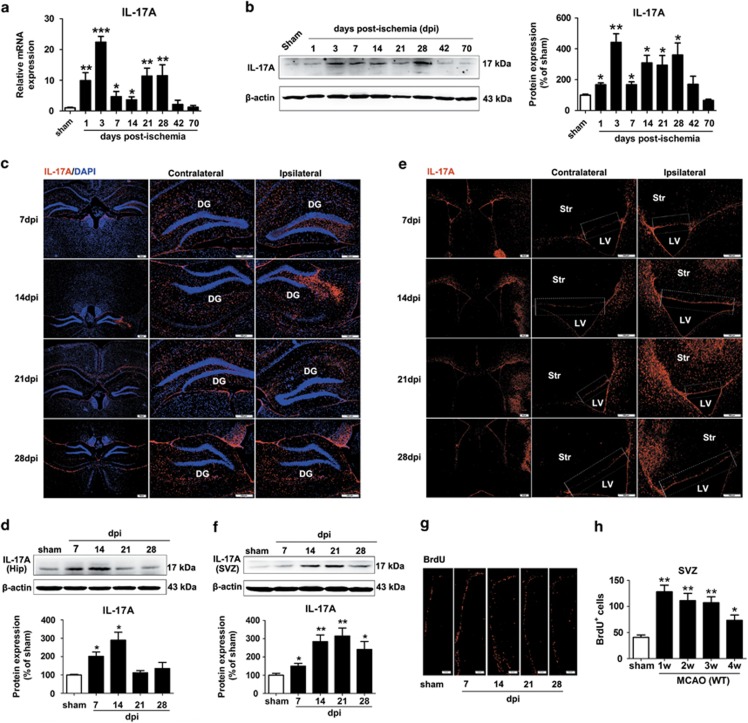
Levels of IL-17 A in brain increased following cerebral ischemic reperfusion. Levels of IL-17 A were measured by real-time PCR (**a**) and western blotting (**b**) in the ischemic hemisphere after 60 min of ischemia and reperfusion for different times. Data represent mean±S.E.M., *n*=5; *P<0.05, **P<0.01, ***P<0.0001, significantly different from sham group. Immunofluorescence images showing cells labeled for IL-17 A (red) in the hippocampal DG (**c**) and SVZ (**e**) at 7, 14, 21 and 28 d.p.i. DAPI was used to label the nuclei. Bar=100 *μ*m. Western blotting for IL-17 A in the ischemic DG (**d**) and SVZ (**f**) at 7, 14, 21 and 28 d.p.i. Data represent mean±S.E.M., *n*=5; *P<0.05, **P<0.01, significantly different from sham group. (**g**) Representative photomicrographs of BrdU immunostaining in the ischemic SVZ from sham-operated mice and ischemic mice at 7, 14, 21 and 28 d.p.i. Bar=50 *μ*m. (**h**) Time course quantification of BrdU-positive cells in the ischemic SVZ after stroke. Data represent mean±S.E.M., *n*=5; *P<0.05, **P<0.01, significantly different from sham group. Hip, Hippocampal; LV, lateral ventricles; Str, striatum

First, we sought to establish the temporal and spatial expression profile of IL-17 A after stroke. We found that IL-17 A mRNA (Figure 1a) and protein expression (Figure 1b) significantly increased on day 1, peaked on day 3 and reached a second peak on day 28 in the ischemic hemisphere. IL-17 A-positive cells were significantly increased at 7 and 14 days post-ischemia (d.p.i.) in the ischemic dentate gyrus (DG), but not at 21 or 28 d.p.i. (Figure 1c); whereas IL-17 A-positive cells were significantly increased at all time points in the SVZ (Figure 1e). The expression profile in the areas was further confirmed by western blotting (Figures 1d and f). Furthermore, IL-17 A levels at different reperfusion time points were parallel to the NPCs proliferation in the ischemic SVZ (Figures 1g and h).

### IL-17 A blockade inhibits, whereas rIL-17 A enhances neuroblasts migration and striatal neurogenesis

**Figure 2:**
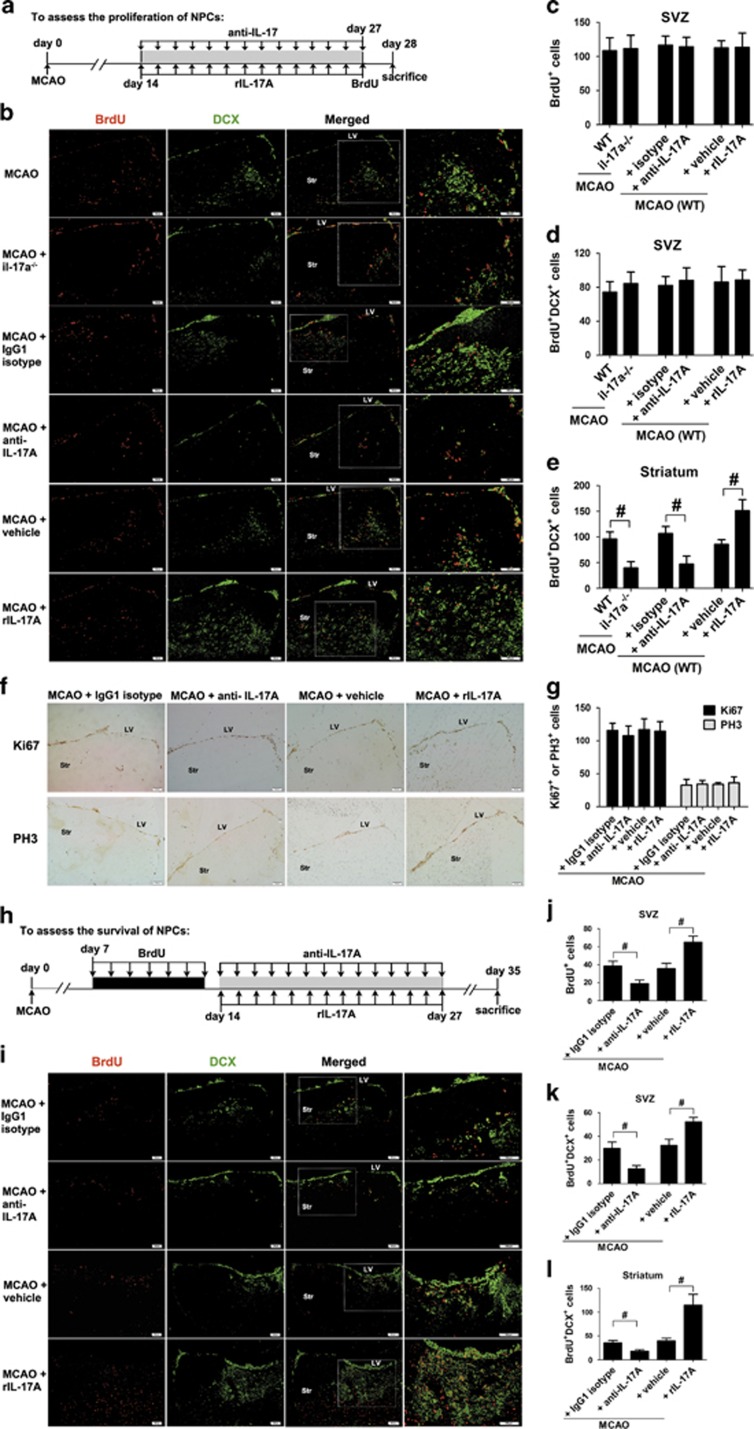
IL-17 A promotes the survival of NPCs and the migration of neuroblasts without affecting the proliferation of NPCs in the SVZ. (**a**) Experimental scheme for assessing cell proliferation in the adult SVZ. (**b**) Immunofluorescence imaging for BrdU^+^ (red) cells coexpressing DCX (green) in the ischemic SVZ and striatum at 28 d.p.i. Bar=100 *μ*m. (**c**) Quantitative comparison of BrdU-labeled cells in the ischemic SVZ for each group. Data represent mean±S.E.M., *n*=5. Quantitative determination of BrdU and DCX double-labeled cells in the ischemic SVZ (**d**) and striatum (**e**) for each group. Data represent mean±S.E.M., *n*=5; ^#^*P*<0.05. (**f**) Representative micrographs of Ki67 and PH3-labeling in the ischemic SVZ at 28 d.p.i. Bar=100 *μ*m. (**g**) Quantitative determination of Ki67 or PH3-labeled cells in the ischemic SVZ for each group. Data represent mean±S.E.M., *n*=5. (**h**) Experimental scheme for assessing cell survival in the adult SVZ. (**i**) Representative images of cells double labeled for BrdU (red) and DCX (green) in the ischemic SVZ and striatum at 35 d.p.i. Bar=100 *μ*m. (**j**) Quantitative comparison of BrdU-labeled cells in the ischemic SVZ for each group. Data represent mean±S.E.M., *n*=5; ^#^*P*<0.05. (**k** and **l**) Quantitative determination of BrdU and DCX double-labeled cells in the ischemic SVZ and striatum for each group. Data represent mean±S.E.M., *n*=5; ^#^*P*<0.05. anti-IL-17 A, anti-IL-17 A monoclonal antibody

We first assessed cell proliferation by treating 27 d.p.i. mice twice with S-phase marker 5-bromo-2′-deoxyuridine (BrdU) with an 8- h interval between injections; on the following day, the animals were humanely killed to analyze BrdU labeling of dividing cells (Figure 2a). Results showed that WT mice treated with recombinant mouse IL-17A (rIL-17 A) or anti-IL-17 A mAb had a similar number of BrdU^+^ cells in the ischemic SVZ to that of WT mice treated with vehicle or isotype at 28 d.p.i. No significant difference in the number of BrdU^+^ cells was observed between IL-17A knock-out (il-17a^−/−^) and WT mice in the ischemic SVZ (Figures 2b and c). Consistently, the number of cell proliferation marker ki67^+^ and M phase marker PH3^+^ cells in the ischemic SVZ was also unaffected in WT mice treated with rIL-17 A or anti-IL-17 A mAb (Figures 2f and g). These data indicate that IL-17 A does not alter the proliferation of SVZ NPCs during stroke recovery.

Next, we evaluated DCX expression because DCX as a marker for neuronal precursors and neurogenesis is expressed by virtually all neuroblasts. DCX^+^ cells are rare within the striatum of non-ischemic or normal control brain.^14, 15^ However, an increase in DCX immunostaining was readily visible in the ischemic striatum at 28 d.p.i. (Figure 2b), indicating that cerebral ischemia stimulates the migration of neuroblasts from the ipsilateral SVZ to the ischemic striatum. Moreover, adult SVZ neurogenesis was detected with BrdU and DCX double marker. BrdU was injected intraperitoneally at 27 d.p.i. to label newly born cells and BrdU/DCX double immunofluorescence was performed at 28 d.p.i., we observed less BrdU^+^/DCX^+^ cells in anti-IL-17 A mAb-treated WT mice or il-17a^−/−^ mice in the ischemic striatum but not the SVZ; rIL-17 A markedly increased the number of BrdU^+^/DCX^+^ cells in the ischemic striatum (Figures 2d and e).

### Promoting effects of IL-17 A on NPCs survival, neuronal differentiation, synaptogenesis and functional recovery

Given IL-17 A had no effect on SVZ NSCs proliferation in adult mice following stroke, then the enhanced striatal neurogenesis induced by IL-17 A could also result from increased NSCs survival.^16, 17^ Therefore, BrdU was given two times daily for 7 days beginning at 7 d.p.i. to determine whether IL-17 A could increase the survival of SVZ NPCs at 35 d.p.i. (Figure 2h). Notably, anti-IL-17 A mAb significantly reduced, whereas rIL-17 A significantly increased the number of BrdU^+^ cells in the ischemic SVZ at 35 d.p.i. (Figures 2i and j), suggesting that IL-17 A does not change proliferating activity but increases the survival of SVZ NPCs.

To determine neuronal identity of BrdU-labeled cells, we examined colocalization of BrdU and DCX at 35 d.p.i. The number of BrdU-labeled neuroblasts in anti-IL-17A mAb-treated mice was significantly reduced in the ischemic SVZ and striatum. In contrast, mice treated with rIL-17A had much more BrdU^+^/DCX^+^ cells (Figures 2k and l).

**Figure 3:**
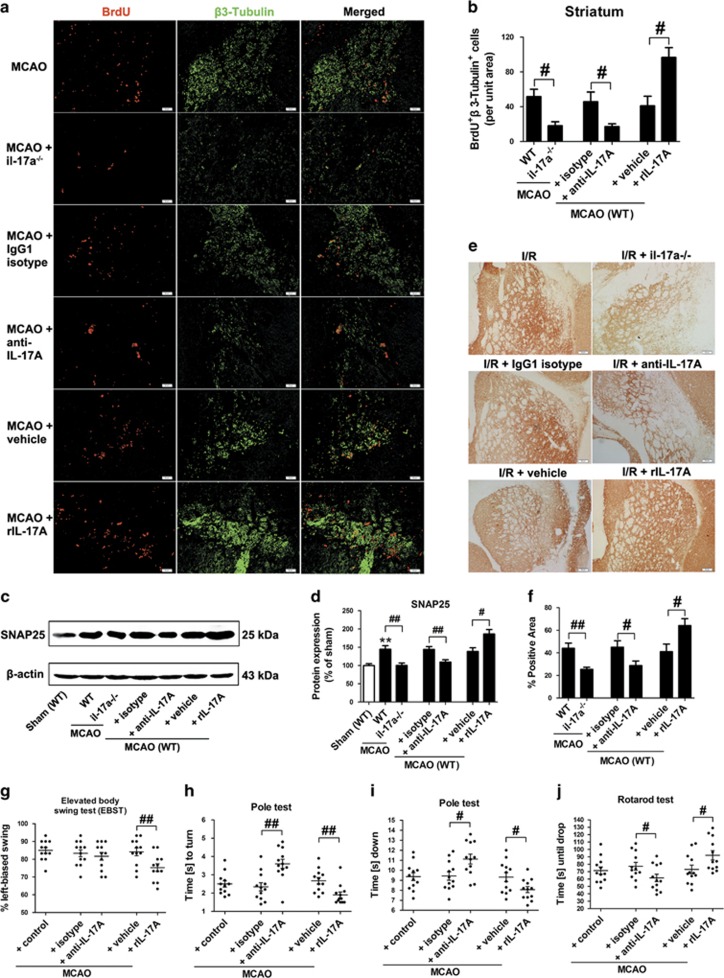
IL-17 A promotes the neuronal differentiation and axonal regeneration, and ultimately improves post-ischemic functional recovery at 35 d.p.i. (**a**) Double immunofluorescence staining with antibodies against BrdU (red) and β3-tubulin (green) in the ischemic striatum. Bar=50 *μ*m. (**b**) Quantitative determination of BrdU and β3-tubulin double-labeled cells in the ischemic striatum for each group. Data represent mean±S.E.M., *n*=5; ^#^*P*<0.05. (**c** and **d**) SNAP-25 western blotting and quantitative data in the IBZ for each group. Data represent mean±S.E.M., *n*=5; **P<0.01, significantly different from sham group; ^#^*P*<0.05, ^##^*P*<0.01. (**e** and **f**) Synaptophysin immunostaining and quantitative data in the IBZ for each group. Data represent mean±S.E.M., *n*=4; ^#^*P*<0.05, ^##^*P*<0.01. Bar=50 *μ*m. Behavioral tests were assessed at 35 d.p.i., including the EBST (**g**), pole test (time to turn completely head down (**h**) and time to reach the floor (**i**)) and rotarod test (**j**). Data represent mean±S.E.M., *n*=12; ^#^*P*<0.05, ^##^*P*<0.01

Not all DCX-positive neuroblasts will convert into neurons. So we further performed double immunofluorescent staining with neuron-specific marker β3-tubulin and BrdU to detect newborn neurons at 35 d.p.i. The number of BrdU^+^/β3-tubulin^+^ cells in rIL-17 A-treated mice was significantly increased in the ischemic striatum. In contrast, il-17a^−/−^ mice or anti-IL-17 A mAb-treated WT mice had much less BrdU^+^/β3-tubulin^+^ cells (Figures 3a and b).

To determine whether the increase in newborn neurons induced by IL-17 A during stroke recovery properly integrates into the striatal network, synaptogenesis markers SNAP-25 and Synaptophysin were used as indices and outcome measures for synaptogenesis at 35 d.p.i.^18, 19^ WT mice showed higher Synaptophysin and SNAP-25 expression in the ischemic boundary zone (IBZ) compared with il-17a^−/−^ mice; anti-IL-17 A mAb treatment also significantly decreased the Synaptophysin and SNAP-25 expression, whereas rIL-17 A conferred the opposite effects (Figures 3c–f).

Improving functional outcome after stroke is the ultimate goal of stroke treatment. We found that deficits in the Pole test (Figures 3h and i) and Rotarod test (Figure 3j) were worse in the anti-IL-17 A mAb-treated WT mice tested at 35 d.p.i. However, the deficits in the elevated body swing test (EBST; Figure 3g), Pole test (Figures 3h and i) and Rotarod test (Figure 3j) were better in the rIL-17 A-treated WT mice. Our data suggest that IL-17 A improves long-term functional recovery.

### IL-17 A promotes neurogenesis and functional recovery via p38 MAPK/calpain 1

**Figure 4:**
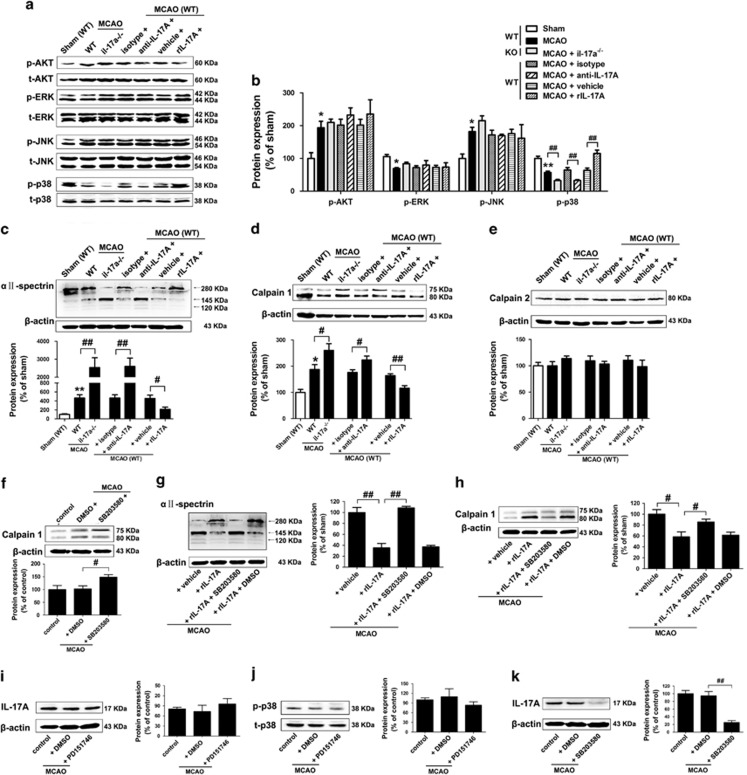
The p38 MAPK pathway is a downstream effector for IL-17 A to inhibit calpain 1 at 28 d.p.i. (**a**) Western blotting for p-Akt, p-ERK, p-JNK and p-p38 in the ischemic hemisphere. (**b**) Quantitative determinations of the levels of p-Akt, p-ERK, p-JNK and p-p38. Data represent mean±S.E.M., *n*=5; *P<0.05, **P<0.01, significantly different from sham group; ^##^*P*<0.01. Western blotting and quantitative data for SBDP145 (**c**), calpain 1 (**d**) and calpain 2 (**e**) in il-17a^−/−^ mice or WT mice treated with rIL-17 A (or vehicle) or anti-IL-17 A mAb (or isotype mAb). Data represent mean±S.E.M., *n*=5; *P<0.05, **P<0.01, significantly different from sham group; ^#^*P*<0.05, ^##^*P*<0.01. (**f** and **k**) Western blotting and quantitative data for calpain 1 and IL-17 A treated with the p38 MAPK inhibitor SB203580. Data represent mean±S.E.M., *n*=5; ^#^*P*<0.05, ^##^*P*<0.01. Western blotting and quantitative data for SBDP145 (**g**) and calpain 1 (**h**) in rIL-17 A (or vehicle) treated mice with or without the addition of SB203580 (or 1% DMSO). Data represent mean±S.E.M., *n*=5; ^#^*P*<0.05, ^##^*P*<0.01. Western blotting and quantitative data for IL-17 A (**i**) and p-p38 (**j**) treated with the calpain 1 inhibitor PD151746. Data represent mean±S.E.M., *n*=5

The finding of IL-17 A on neurogenesis and functional recovery compelled us to further explore IL-17 A-activated downstream signaling pathways. MAPK and PI3K/Akt are major signaling pathways implicated in NPCs proliferation and differentiation.^20, 21, 22, 23, 24^ As the most abundant calpain molecules in the brain,^25^ calpain 1 maintains stemness and represses neural differentiation of NPCs, whereas calpain 2 acts as potential modulator of gliogenesis *in vitro*.^26^ We thereby detected the expression of these proteins at 28 d.p.i. The ratio of 76 : 80 kD reflects calpain 1 activity.^27^ IL-17 A deficiency or anti-IL-17 A mAb treatment significantly reduced p-p38 levels without altering the levels of p-ERK, p-JNK and p-AKT (Figures 4a and b). Furthermore, IL-17 A deficiency or anti-IL-17 A mAb treatment significantly increased the levels of calpain-specific aII-spectrin breakdown products of 145 kDa (SBDP145) (Figure 4c) and the ratio of 76 kD/80 kD of calpain 1 (Figure 4d), but not calpain 2 at 28 d.p.i. (Figure 4e). In contrast, rIL-17 A treatment significantly increased p-p38 levels, and significantly reduced the levels of SBDP145 and the ratio of 76 kD/80 kD of calpain 1 (Figures 4a–e). These results indicate that both p38 MAPK and calpain 1 are the downstream signal molecules for IL-17 A.

MAPK has previously been implicated in the regulation of calpain activity,^28, 29^ thus we sought to explore the intrinsic link between p38 MAPK and calpain 1 in our stroke recovery model. SB203580 significantly increased the ratio of 76 kD/80 kD of calpain 1 (Figure 4f). The inhibitory effects of IL-17 A on SBDP145 expression and calpain 1 activity were significantly blocked by SB203580 (Figures 4g and h). However, calpain 1 inhibitor PD151746 had no effect on the levels of IL-17 A and p-p38 (Figures 4i and j). Our results suggested that p38 MAPK is a downstream signal molecule for IL-17 A to inhibit calpain 1 in our stroke recovery model.

**Figure 5:**
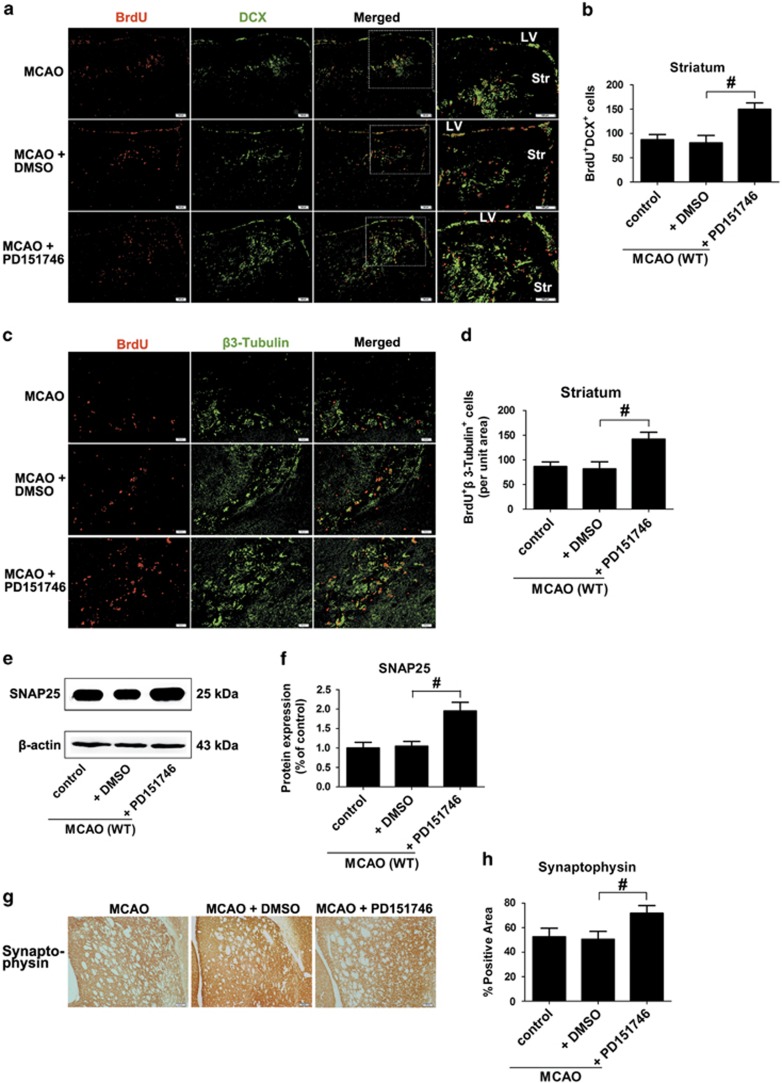
Calpain 1 inhibits neuroblasts migration toward the ischemic lesion, neuronal differentiation and synaptogenesis in adult mice at 35 d.p.i. (**a**) Representative images of cells double labeled for BrdU (red) and DCX (green) in the ischemic striatum. Bar=100 *μ*m. (**b**) Quantitative determination of BrdU and DCX double-labeled cells in the ischemic striatum for each group. Data represent mean±S.E.M., *n*=5; ^#^*P*<0.05. (**c**) Representative images of cells double labeled for BrdU (red) and β3-tubulin (green) in the ischemic striatum. Bar=50 *μ*m. (**d**) Quantitative determination of BrdU and β3-tubulin double-labeled cells for each group. Data represent mean±S.E.M., *n*=5; ^#^*P*<0.05. (**e** and **f**) SNAP-25-western blotting and quantitative data in the IBZ for each group. Data represent mean±S.E.M., *n*=5; ^#^*P*<0.05. (**g** and **h**) Immunohistochemistry labeling and quantitative analyses for Synaptophysin in the IBZ for each group. Data represent mean±S.E.M., *n*=5; ^#^*P*<0.05. Bar=50 *μ*m

Next, we investigated the function of calpain 1 in IL-17 A-induced enhancement of neurogenesis and functional recovery. PD151746 significantly increased the number of BrdU^+^/DCX^+^ (Figures 5a and b) and BrdU^+^/β3-tubulin^+^ cells (Figures 5c and d) in the ischemic striatum compared with the DMSO-treated control group at 35 d.p.i. The SNAP-25 (Figures 5e and f) and Synaptophysin levels (Figures 5g and h) in the IBZ were also significantly increased by the treatment with PD151746, indicating a dominant-negative effect of calpain 1 on neurogenesis and synaptogenesis during stroke recovery.

### Astrocytes are the major cellular source of IL-17 A during stroke recovery

**Figure 6:**
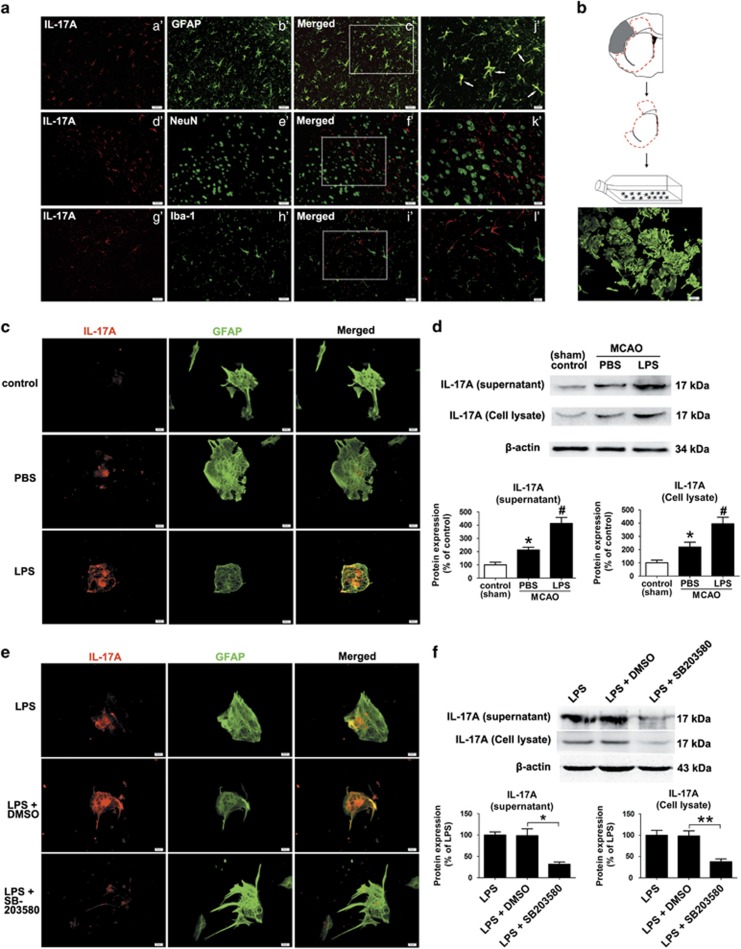
Astrocytes are the major cellular source of IL-17 A in the recovery phase of stroke, and reactive astrocytes secrete IL-17 A in a p38 MAPK-dependent manner. (**a**) *In vivo* immunofluorescence images showing cells double labeled with anti-IL-17 A (red) and anti-GFAP, anti-NeuN, or anti-Iba-1 (green) at 28 d.p.i. Bar=a'–i'=50 *μ*m, j'–l'=20 *μ*m. (**b**) Representative schematic drawing showing the isolation of SVZ astrocytes from ischemic brains at 14 d.p.i. (**c**) Representative fluorescence images of primary cells stained with IL-17 A (red) and GFAP (green) in cultured mouse astrocytes treated with LPS or 1% DMSO. Bar=20 *μ*m. (**d**) Western blotting and quantitative data for IL-17 A in the culture supernatant or in the cell lysates of LPS-activated mouse astrocytes. Data represent mean±S.E.M. from three experiments; **P<0.01, significantly different from control group; ^#^*P*<0.05, significantly different from PBS treatment group. (**e**) Fluorescence images of primary cells double labeled for IL-17 A (red) and GFAP (green) in LPS-activated mouse astrocytes treated with SB203580 or 1% DMSO. Bar=20 *μ*m. (**f**) Western blotting and quantitative data for IL-17 A in the culture supernatant or in the cell lysates of LPS-activated mouse astrocytes treated with SB203580 or 1% DMSO. Data represent mean±S.E.M. from three experiments; *P<0.05, **P<0.01

To our surprise, SB203580 treatment significantly reduced IL-17 A levels at 28 d.p.i. (Figure 4k). To elucidate the mechanisms underlying the crosslink between IL-17 A and p38 MAPK, double immunofluorescence labeling was performed to identify which cells were responsible for the elevation of IL-17 A at 28 d.p.i. IL-17 A immunoreactivity was colocalized with the astrocyte marker glial fibrillary acidic protein (GFAP) but not with the microglia marker Iba-1 or the neuronal marker NeuN, indicating that astrocytes are the main cellular source of IL-17 A in our stroke recovery model (Figure 6a). Furthermore, in primary astrocytes cultures from the ischemic mice at 14 d.p.i. (Figure 6b), astrocytes were stimulated with lipopolysaccharide (LPS) to mimic a reactive phenotype. We observed a significant increase of IL-17 A in both the cell lysate and culture supernatant of astrocytes treated with LPS (Figures 6c and d). Inhibiting p38 MAPK by the administration of SB203580 significantly blocked the promoting effect of LPS on the production and secretion of IL-17 A (Figures 6e and f). These data suggest that the p38 MAPK pathway is essential for the secretion of IL-17 A from astrocytes.

### IL-17 A promotes the neuronal differentiation of NPCs *in vitro*

**Figure 7:**
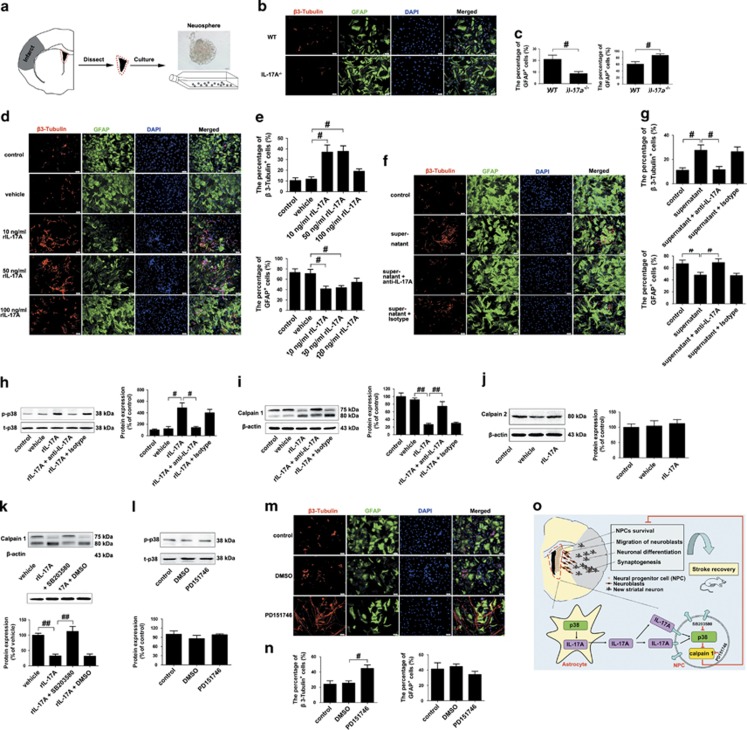
IL-17 A promotes neuronal differentiation of NPCs via p38 MAPK/calpain 1 *in vitro*. (**a**) Representative schematic drawing showing the isolation of SVZ NPCs from ischemic brains at 14 d.p.i. Bar=20 *μ*m. Double immunofluorescence labeling and quantitative analyses for β3-tubulin (red) and GFAP (green) in passage 2 NPCs dissociated from WT or il-17a^−/−^ mice (**b** and **c**), or in passage 2 NPCs treated with different concentrations of IL-17 A (**d** and **e**) or the culture supernatant from reactive astrocytes (**f** and **g**) after 7 days of differentiation. DAPI was used to label the nuclei. Data represent mean±S.E.M. from three experiments; ^#^*P*<0.05. Bar=50 *μ*m. Western blotting and quantitative data for p-p38 (**h**), calpain 1 (**i**) and calpain 2 (**j**) in passage 2 NPCs treated with rIL-17 A (or vehicle) with or without the addition of anti-IL-17 A mAb (or isotype mAb) after 7 days of differentiation. Data represent mean±S.E.M. from three experiments; ^#^*P*<0.05, ^##^*P*<0.01. Western blotting and quantitative data for calpain 1 in passage 2 NPCs treated with rIL-17 A (or vehicle) with or without the addition of SB203580 or 1% DMSO (**k**), or for p-p38 in passage 2 NPCs treated with PD151746 or 1% DMSO (**l**). Data represent mean±S.E.M. from three experiments; ^##^*P*<0.01. (**m** and **n**) Double immunofluorescence labeling and quantitative analyses for β3-tubulin (red) and GFAP (green) in passage 2 NPCs treated with PD151746 or 1% DMSO. Data represent mean±S.E.M. from three experiments; ^#^*P*<0.05. Bar=50 *μ*m. (**o**) A working model: IL-17 A promotes functional recovery through enhancing NPCs survival, neuroblasts migration, neuronal differentiation and synaptogenesis through p38 MAPK/calpain 1 signaling pathway

To further investigate the direct function of IL-17 A in NPCs, we prepared neurosphere cultures from the SVZ of ischemic mice at 14 d.p.i. (Figure 7a). We analyzed the role of IL-17 A activation in NPCs differentiation, immunofluorescent staining with antibodies against β3-tubulin and GFAP was performed on NPCs after 7 days of differentiation. As expected, NPCs from il-17a^−/−^ mice differentiated into fewer β3-tubulin^+^ neurons (Figures 7b and c). However, treatment with 10 or 50 ng/ml rIL-17 A significantly increased the percentage of β3-tubulin^+^ neurons and reduced the percentage of GFAP^+^ astrocytes (Figures 7d and e). After 16-h treatment with LPS, the culture supernatant of reactive astrocytes was added to the differentiation medium beginning 2 h after differentiation induction every 24 h for 7 days. We observed a significant increase in the percentage of β3-tubulin^+^ neurons; when anti-IL-17 A mAb was added, the effects of the supernatant on NPCs differentiation into neurons were significantly blocked (Figures 7f and g). These data indicate that IL-17 A leads to increased neuronal differentiation but decreased astrocyte differentiation of NPCs *in vitro*.

We then explored the potential underlying mechanisms *in vitro*. Passage 2 SVZ neurospheres were dissociated to single cells and incubated in the differentiation medium. Then, cells were treated with 50 ng/ml rIL-17 A or 2 *μ*g/ml anti-IL-17 A mAb 2 h after differentiation induction and harvested for western blotting analysis after another 24 h. Treatment with rIL-17 A significantly increased the levels of p-p38 (Figure 7h) and significantly reduced the ratio of 76 kD/80 kD of calpain 1 (Figure 7i) without affecting the expression of calpain 2 (Figure 7j). When anti-IL-17 A mAb was added, the effects of rIL-17 A on the p-p38 expression or calpain 1 activity were significantly blocked (Figures 7h and i). Pre-treatment with 10 *μ*M SB203580 30 min before the addition of rIL-17 A significantly blocked the inhibitory effect of rIL-17 A on calpain 1 activity (Figure 7k). In all, 40 *μ*M PD151746 had no effect on the expression of p-p38 (Figure 7l). Thus, these data show that p38 MAPK is a downstream signal molecule for IL-17 A to inhibit calpain 1 in NPCs *in vitro*.

We also found that 7 days treatment with PD151746 significantly increased the number of β3-tubulin^+^ neurons (Figures 7m and n). Taken together, these results implicate that IL-17 A promotes neural differentiation of NPCs derived from the SVZ of ischemic mice via p38 MAPK/calpain 1.

## Discussion

The IL-17 family consists of a couple of cytokines that participate in both acute and chronic inflammatory responses.^30^ IL-17 A is the most widely investigated cytokine of this family where its production has been mainly attributed to T helper 17 cells.^30^ Recent studies have revealed that IL-17 A is mainly produced by γδ T cells in the acute phase of stroke. Our and other previous studies have shown that IL-17 A, whose expression peaks on day 3 after stroke, mediates innate immune response and contributes to acute ischemic brain injury.^6, 31^ In this study, two peaks of IL-17 A expression levels were demonstrated at 3 and 28 d.p.i., corresponding, respectively, to the acute phase of stroke and later phases of recovery stroke. The appearance of a very moderate expression of IL-17 A in the ischemic brain at day 7 may foreshadow this second expression of IL-17 A after stroke. Our data showed for the first time that the delayed elevation of IL-17 A improved brain tissue repair and spontaneous recovery after stroke, as demonstrated by our data that blocking IL-17 A with IL-17 A neutralizing antibody or IL-17 A deficiency markedly inhibited ischemia-induced spontaneous recovery through reducing SVZ NPCs survival, migration of newly born neuroblasts into the ischemic striatum, neuronal differentiation and subsequent synaptogenesis. Endogenous neurogenesis may be one of the mechanisms underlying the spontaneous recovery after stroke, but its contribution is probably minor.^32^ Our further results showed that intranasally administration of rIL-17 A conferred the opposite effects. Therefore, exogenous intervention by upregulating IL-17 A levels in the recovery phase could further promote ischemia-induced neurogenesis and long-term functional recovery.

Our data demonstrated that IL-17 A was the critical requisites for SVZ NPCs survival but not their proliferation. Blocking IL-17 A reduced, whereas intranasally administration with rIL-17 A increased SVZ NPCs survival at 35 d.p.i., when S-phase marker BrdU was given for 1 week starting at 7 d.p.i. to assess cell survival.^16, 17^ However, rIL-17 A treatment or blocking IL-17 A did not alter SVZ NPCs proliferation, when BrdU was given at 27 d.p.i. and BrdU labeling was analyzed at 28 d.p.i. This notion was further supported by our observations that IL-17 A did not change the expression of the cell proliferation markers Ki67 and PH3 in the SVZ.^33, 34^ In this study, blocking IL-17 A reduced, whereas intranasally administration with rIL-17 A increased the number of BrdU^+^/DCX^+^ cells in the ischemic striatum, indicating that the increase in the survival of SVZ NPCs mediated by IL-17 A could promote the migration of neuroblasts toward the ischemic striatum. Moreover, the increased survival of SVZ NPCs could also increase the neuronal differentiation (BrdU^+^/β3-tubulin^+^ cells) of NPCs in the ischemic striatum. Thus, our data indicated that IL-17 A may be critical for ischemia-induced migration of newly born neuroblasts and differentiation into neurons because it facilitates the survival of NPCs. To date, the newly generated neurons mediated by IL-17 A during stroke recovery need to form synapses and functionally integrate into the striatal network, and contribute to restoring damaged neuronal networks so as to promote long-term functional recovery.^35, 36^ We found that blocking IL-17 A inhibited synaptogenesis, whereas intranasal administration of rIL-17 A promoted synaptogenesis, which foster improved functional recovery after stroke. Together, our results show that IL-17 A secreted from reactive astrocytes acts directly on SVZ NPCs and promotes stroke recovery through increasing SVZ NPCs survival, migration of neuroblasts, neuronal differentiation and subsequent synaptogenesis (Figure 7o).

Apart from γδ T cells, other cells, including NK cells and resident astrocytes can also produce IL-17 A in brain tissue.^11, 37, 38^ In this study, we showed for the first time that IL-17 A was predominantly secreted by reactive astrocytes, which can act as antigen-presenting cells (APCs) in neuroinflammation.^39, 40^ One previous study indicates the role of astrocytes as the resident immune effector cells in the recovery phase of stroke, as demonstrated by the finding that reactive astrocytes can release an immunomodulatory cytokine called high-mobility group box 1 (HMGB1), which is also able to activate APCs and stands at the crossroads of innate and adaptive immunity,^41^ promotes endothelial progenitor cell-mediated neurovascular remodeling and functional recovery.^11^ Our present results further expand our understanding of the important role of astrocytes as immunocompetent effector cells during stroke recovery, which could be clinically exploited. Further studies should be done to clarify the immune competent role of astrocytes after ischemic stroke.

Our and other previous studies have shown that immune activation is essential for the pathologic activation of calpain,^7, 42^ as demonstrated by our previous finding that elevated IL-17 A induced by ischemia activates calpain in the acute phase of stroke.^31^ Importantly, calpain activity is shown to be modulated during neural differentiation of rat pheochromocytoma cells.^43, 44^ Furthermore, one *in vitro* study have shown that calpain 1 maintains stemness and represses neuronal differentiation of NPCs, whereas calpain 2 acts as potential modulator of gliogenesis.^26^ Based on these findings, we explored the intrinsic link between immune activation and calpain activity in IL-17 A-induced neurogenesis in the later phases of stroke recovery. In this study, our *in vivo* and *in vitro* data showed that IL-17 A maintained and augmented ischemia-induced neurogenesis through the inhibition of calpain 1 activity during stroke recovery, which suggests an essential role of calpain 1 in IL-17 A-mediated neurogenesis after stroke. Furthermore, our results showed that p38 MAPK also have important role in IL-17 A-mediated neurogenesis after screening for PI3K/Akt and MAPK signaling pathways, which are major signaling pathways implicated in the proliferation and differentiation of NPCs.^20, 21, 22, 23, 24^ These data indicate that both p38 MAPK and calpain 1 are the downstream signal molecules for IL-17 A and have important roles in IL-17 A-mediated neurogenesis during stroke recovery. As previously reported, the MAPK signaling pathways are implicated in the regulation of calpain activity.^28, 29^ This study showed that IL-17 A-induced inhibition of calpain 1 activity is dependent on p38 MAPK signaling. Together, we provided *in vivo* and *in vitro* evidences that IL-17 A promoted neurogenesis via p38 MAPK/calpain 1 signaling pathway.

In summary, our results indicate that IL-17 A may have biphasic role in ischemic stroke. IL-17 A from γδ T cells worsens acute brain injury in the acute stage of stroke,^7^ whereas IL-17 A from astrocytes maintains the survival and neuronal differentiation of NPCs, and subsequent synaptogenesis and spontaneous recovery via p38 MAPK/calpain 1 signaling pathway in the delayed phases of stroke recovery. In conclusions, our results reveal a previously uncharacterized property of astrocytic IL-17 A in the endogenous neurorepair and spontaneous recovery after ischemic stroke.

## Materials and Methods

### Materials

rIL-17 A was purchased from R&D Systems (Minneapolis, MN, USA). Anti-IL-17 A monoclonal antibody (mAb) and mouse IgG1 isotype control mAb (isotype) were purchased from eBioscience (San Diego, CA, USA). SB203580 was purchased from MedChem Express (Monmouth Junction, NJ, USA). PD151746, insulin–transferrin–selenium (ITS), and poly-l-ornithine were purchased from Sigma Chemicals (St. Louis, MO, USA). Epidermal growth factor (EGF) and basic fibroblast growth factor (bFGF) were purchased from PeproTech (Rocky Hill, NJ, USA). Penicillin plus streptomycin was from Beijing Solarbio Science and Technology (Beijing, China). Heparin was purchased from StemCell Technologies (Vancouver, BC, Canada). B27 supplement, laminin and FBS were purchased from Invitrogen/Gibco (Carlsbad, CA, USA). DMEM/F-12 medium was from Thermo Scientific HyClone (Logan, UT, USA). Antibodies against mouse synaptosomal-associated protein-25 kDa (SNAP-25), phosphor (p)-AKT (p-AKT), t-AKT, p-extracellular regulated protein kinases (p-ERKs), t-ERK, phospho-c-Jun N-terminal kinases (p-JNKs), t-JNK, p-p38, t-p38, calpain 1, calpain 2, BrdU, doublecortin (DCX), Ki67, phospho-histone H3 (PH3), β3-tubulin, Synaptophysin, GFAP and NeuN were purchased from Cell Signaling Technology (Beverly, MA, USA). Antibodies against mouse IL-17 A and aII-spectrin were purchased from Abclonal Technology (Shanghai, China). Antibodies against mouse Iba-1 were purchased from Wako Pure Chemical Industries (Osaka, Japan).

### Mice

Male wild-type (WT) C57BL/6 mice were purchased from Wuhan University Laboratory Animal Center (Wuhan, China). Male il-17a^−/−^ mice (on a C57BL/6 background) were kindly provided by Y Iwakura (University of Tokyo, Tokyo, Japan). Mice used for all experiments were 8–10 weeks old and were housed under specific pathogen-free conditions at Animal Laboratory Center of Tongji Medical College.

### Middle cerebral artery occlusion (MCAO) model of transient focal ischemia

All experiments with mice were performed in accordance with protocols approved by the Animal Care and Use Committee of Tongji Medical College, Huazhong University of Science and Technology. C57BL/6 mice (24–25 g) or il-17a^–/–^ mice (24–25 g) were anesthetized i.p. with ketamine (100 mg/kg) and xylazine (8 mg/kg). Focal cerebral ischemia was induced by MCAO with a 6–0 silicone-coated nylon monofilament, as previously described.^31^ Occlusion was confirmed by laser-Doppler flowmeter (Periflux system 5000, PERIMED, Stockholm, Sweden) with a probe placed on thinned skull over the lateral parietal cortex.^31^ After 60 min of occlusion, the filament was removed and reperfusion was verified. Body temperature was maintained at 37±0.5 °C with a feed-back temperature control unit until the mice had recovered from surgery. Our previous studies showed no physiological differences between groups.^6, 31^

### Drug treatments

Treatment groups were assigned in a randomized and blinded manner. In all, 2 *μ*g anti-IL-17 A mAb or 2 *μ*g mouse IgG1 isotype control mAb was injected i.c.v. into the left lateral ventricle (0.9 mm laterally, 0.1 mm posteriorly, 3.1 mm deep from the bregma). A sterile 26-G Hamilton microsyringe (80330; Hamilton Company, Reno, NV, USA) was used to intranasally administer 2 *μ*l drops of rIL-17 A diluted in PBS containing 0.1% albumin (0.1 *μ*g/*μ*l) or its vehicle (PBS containing 0.1% albumin) to alternating nostrils with a 2-min interval between applications. Drops were placed at the opening of the nostril, allowing the mice to snort each drop into the nasal cavity. A total of 10 *μ*l of dose solution, containing 1 *μ*g rIL-17 A was delivered over a course of 5 min. The injection of anti-IL-17 A mAb (or isotype) or rIL-17A (or vehicle) was repeated every 24 h for 2 week starting at 14 d.p.i.

SB203580 was dissolved in 1% DMSO at 1 mg/ml. Mice were injected i.p. with SB203580 (10 mg/kg) or 1% DMSO daily for 14d, starting at 14 d.p.i. or 30 min before the application of rIL-17 A. PD151746 was dissolved in 1% DMSO at 0.8 mg/ml. PD151746 (0.2 mg/kg) was injected i.c.v. daily for 2 week, starting at 14 d.p.i.

### BrdU labeling

We first assessed cell proliferation by treating mice twice with BrdU (dissolved at 10 mg/ml in saline, 50 mg/kg per injection; Sigma, St. Louis, MO, USA) with an 8-h interval between injections at 27 d.p.i.; on the following day, the animals were humanely killed to analyze BrdU labeling of dividing cells. In the second experiment, BrdU was given i.p. two times daily for 1 week starting at 7 d.p.i. BrdU labeling was analyzed at 35 d.p.i. to assess cell survival.^16, 17^

### Functional assays

The EBST was used to test asymmetrical motor behavior. The rotarod test and pole test were used to assess locomotor balance and coordination. EBST and rotarod test were performed as described at 35 d.p.i.^45^ The pole test was performed as described previously at 35 d.p.i.^46^

### Neurosphere cultures

Ipsilateral SVZ cells were dissociated from ischemic hemispheric brains at 14 d.p.i. After digestion, the cells were cultured in cell proliferation medium (DMEM/F-12 medium supplemented with 1% penicillin plus streptomycin, 2 *μ*g/ml heparin, 1% ITS, 2% B27 supplement, 20 ng/ml EGF and 20 ng/ml bFGF) at a concentration of 5 × 10^4^ cells/ml. Cells were maintained at 37 °C with 5% CO_2_ in an incubator with high humidity. The medium was changed twice per week. After 7–10 days of culture, primary neurospheres of 100–150 *μ*m in diameter could be collected without disturbing the attached cells and were passaged into fresh cell proliferation medium. Passage 2 SVZ neurospheres were dissociated to single cells and placed onto a poly-l-ornithine- and laminin-coated coverslip in cell proliferation medium. At 18-h post-plating, medium was changed into differentiation medium (DMEM/F-12 medium supplemented with 1% penicillin plus streptomycin, 1% ITS, 2% B27 supplement, 1% FBS) and differentiated for 7 days.

### Assessment of NPCs differentiation

At 2 h after differentiation induction, the differentiation medium of passage 2 neurospheres was supplemented with rIL-17 A (10, 50 or 100 ng/ml) or its vehicle, and/or with anti-IL17 A neutralized mAb (2 *μ*g/ml) or isotype every 24 h for 7 days. Differentiated cells were collected by either direct cell lysis for western blotting analysis or by fixation using 4% paraformaldehyde for immunohistochemical staining using cell lineage-specific antibodies.

### Primary astrocyte cultures

Cells were dissociated from ischemic hemispheric brains at 14 d.p.i. or sham-operated brains as described before.^47^ Briefly, dissociated cells were seeded into poly-l-lysine-coated 25 cm^2^ flasks and cultured in DMEM/F-12 medium supplemented with 1% penicillin plus streptomycin (10 000 U/ml), 10% FBS (complete medium). The culture medium was renewed every 3 days. After approximately 7 days, non-astrocytic cells, such as microglia and neurons, were detached from the flasks by shaking at 180 revolutions per minute at 37 °C for 18 h and removed by changing the medium. The adherent cells were trypsinized, resuspended in complete medium for 30 min and plated in the flasks (5 × 10^5^ cells/ml). The cells were purified by repeated trypsinization and inoculated at a density of 5 × 10^5^ cells/ml at least four times until >95% of the cultured cells were astrocytes as identified by immunofluorescent staining for GFAP.

### *In vitro* LPS stimulation and treatments in the astrocyte culture

Purified astrocytes were treated with LPS (100 ng/ml; Sigma) in the presence or absence of pretreatment with SB203580 (10 *μ*M, 30 min before LPS treatment). At 16 h after treatment, the cells were fixed with 4% paraformaldehyde and subjected to histological analysis. The supernatant of cell culture was collected for western blotting analysis.

### Western blotting

Western blotting was done according to our previously established protocols.^6, 31^ Tissue samples or primary cells were lysed in RIPA buffer containing protease and phosphatase inhibitors (KeyGen Biotech Co., Ltd, Nanjing, China). Cell culture supernatants proteins were extracted as previously described.^48^ Equal amounts of protein were subjected to SDS-PAGE analysis, transferred onto PVDF membrane and probed with primary antibodies against IL-17 A, SNAP-25, p-AKT, total-AKT, p-ERK, t-ERK, p-JNK, t-JNK, p-p38, t-p38, aII-spectrin, calpain 1 and calpain 2. After washing, the membranes were treated with the corresponding HRP-conjugated secondary antibody. Chemiluminescence detection was carried out with ECL Western Blotting Detection Reagents (Millipore, Billerica, MA, USA) plus BioWest enhanced chemiluminescence (UVP, Upland, CA, USA). Band intensity was quantified with Image J software (National Institutes of Health, Bethesda, MD, USA).

### Immunocytochemistry

Mice were transcardially perfused with 4% paraformaldehyde, and the brains were embedded with paraffin. Brains were cut into 4-*μ*m-thick coronal sections. Cell cultures were fixed using 4% paraformaldehyde for 15 min. For both sections and cells, nonspecific binding was blocked using normal goat serum. Immunoassays were performed using the following antibodies at concentrations (and using protocols) recommended by the respective manufacturers: anti-IL-17 A, anti-BrdU, anti-DCX, anti-Ki67, PH3, β3-tubulin, Synaptophysin, GFAP, NeuN and Iba-1. In immunofluorescence, primary antibodies were detected with cy3-conjugated goat anti-rabbit, dylight 488-conjugated goat anti-mouse or dylight 488-conjugated goat anti-rat secondary antibody. The nuclei were counterstained by DAPI. In immunohistochemistry, the biotinylated secondary antibody, avidin-biotinylated enzyme complex (ABC) and DAB substrate were used as the detecting reagents (Zhongshan Goldenbridge Biotechnology, Beijing, China). Images were acquired from five random slides each brain, with each slide containing eight fields view within the cortex and striatum. The percentage of Synaptophysin in the IBZ was analyzed with a Micro Computer Imaging Device (MCID) imaging analysis system (Imaging Research, St. Catharines, Canada).

### Real-time PCR analysis

Total RNA preparation and real-time PCR were performed according to our previously established protocols.^31^

### Statistical analyses

Multiple comparisons were performed by one-way ANOVA followed by Newman–Keuls multiple comparison tests for multiple comparisons (GraphPad Prism statistics software version 5.0, La Jolla, CA, USA). Two groups were compared by two-tailed Student's *t*-test. Behavioral data were analyzed by two-way ANOVA with repeated measures, followed by *post hoc* multiple comparison tests. All data are presented as mean±S.E.M. The *P-*values <0.05 were considered statistically significant.

## Abbreviations

IBZ: ischemic boundary zone
IL-17 A: interleukin-17 A
γδ T: gamma delta T cells
SVZ: subventricular zone
NPCs: neural precursor cells
MAPK: mitogen-activated protein kinase
ITS: insulin–transferrin–selenium
EGF: epidermal growth factor
bFGF: basic fibroblast growth factor
ERK: extracellular regulated protein kinase
JNK: c-Jun N-terminal kinase
SNAP-25: synaptosomal-associated protein-25 kDa
DCX: doublecortin
PH3: phospho-histone H3
GFAP: glial fibrillary acidic protein
il-17a^−/−^: IL-17 A knock-out
WT: wild-type
MCAO: middle cerebral artery occlusion
BrdU: 5-bromo-2′-deoxyuridine
EBST: elevated body swing test
LPS: lipopolysaccharide
DG: dentate gyrus
SBDP145: aII-spectrin breakdown products of 145 kDa
HMGB1: high-mobility group box 1
